# Mechanosensitive Ion Channels in Cardiovascular Physiology

**Published:** 2014-10-29

**Authors:** Jinfeng Teng, Steve Loukin, Ching Kung

**Affiliations:** 1 Laboraotry of Cell and Molecular Biology, University of Wisconsin – Madison, WI 53706, USA; 2 Department of Genetics, University of Wisconsin – Madison, WI 53706

**Keywords:** Mechanosensitive (MS) channels, stretch-activated channels, TRPs, K_2p_

## Abstract

EC coupling is subjected to a mechanical feedback, which originates from physical force-sensing ion channels in the pericardium and elsewhere. Reviewed here are the most recent developments that greatly advanced our understanding of these mechanosensitive (MS) channels, including TRPs and K_2p_’s. Patch clamp has continued to demonstrate the direct channel activation by membrane stretch. Crystallography and cryo-electron microscopy have revealed the structures of several MS channels at atomic resolution. Some have been purified to homogeneity, reconstituted into lipid bilayer, and still retain their ability to respond to stretch force. A force-from-lipid (FFL) theory has been advanced that emphasizes the strong binding between channel proteins and lipids. Through these bonds, the sharp lateral tension (akin to surface tension) of the bilayer can transmit added force to the channel protein. Like temperature sensitivity, sensitivity to mechanical force is far more pervasive than we previously realize, and is especially important to the beating heart.

## 1. Introduction

The beating heart is the most obvious mechanical organ. Each systolic-diastolic cycle entails large pressure stress and tissue strain. Its apparent clockwork should not obscure the sophistication of its intricate internal regulations. Each beat generates a force cycle, which is capable of influencing future beats. The electric and Ca^2+^ events in pace making and EC coupling are more thoroughly understood and thus emphasized in current cardiology literature. However, the physical stretching or shortening, in turn, controls the electrophysiological events, forming an arm of the mechano-electric feedback (MEF) loop [1] ^,^[2,3]. Over-stretching the atrium reshapes the action potential, causing arrhythmia [4]. Mechanical aberrations such as ventricular volume or pressure overload can also lead to arrhythmias [1], and myocardial hypertrophy as a maladaptive process occurring in response to an increased cardiac workload [5] [6] [7] Conversely, a single fist blow at the sternum (precordial thump) can sometime restore the normal cardiac rhythm to an arrhythmic patient. Though less is known about the MEF arm of the feedback because of experimental difficulties in the past, recent technical and conceptual advances promise a much deeper understanding. These includes structures at atomic resolutions of heart-relevant MS channel proteins and experiments that support a unifying force-from lipid biophysical theory. We will summarize these new findings below. The muscle hardware has a build-in response to stretch. A longitudinally stretched myocyte produces proportionally stronger contractive force. This is partly explained by reactions of the force generators (actomysin and titin) themselves and can be observed after chemical permeabilization of the membrane [8]. However, in preparations with intact membranes, stretch also generates electrophysiological effects that constitute MEF. An increase in rat atrial pressure strengthens the Ca^2+^ transient and hastens its decay. Myocyte stretching alters the duration of the action potential. These effects require the activation of MS channels [9]. See [10]^,^ [11]^,^ [3] and [12] for recent reviews on MEF.

## 2. Mechanosensitive (MS) ion channels

A mechanosensitive (MS) channel is a transmembrane protein, which uses external mechanical force to bias its open probability and therefore the amount of ions it lets through. The transduction is direct, *i.e.* the MS-channel protein itself receives the stretch force and not, say, receives a ligand produced by a force-sensing enzyme. Though the physiological and pathological concerns are on the whole heart, an intact beating organ makes difficult the investigations with fragile glass electrodes. Reduced systems, such as multicellular heart tissues hold promise [3]. To date, clearer insights comes from greatly reduced systems such as isolated cardiac myocytes or from identified MS channels heterologously expressed in cultured non-cardiac cells for molecular investigation. Early on, unitary cation conductances were recorded under patch clamp that increase their open probability when a suction was applied to stretch the patches of chick embryonic or guinea-pig cardiomyoctes [13]. Similarly, patch clamp revealed in rat atrial cells a cation unitary conductance capable of passing Ca^2+^ that can be activated by pipet suction or pressure [14]. This preparation also revealed a suction induced K^+^ conductance, which is also activated by arachidonic acid and other lipophilic compounds [15], a hallmark feature of some characterized MS channels that respond directly to lipid forces. However, the molecular identities that underlie these cardiomyocyte conductances are unknown. On the other hand, an increasing number of channels of known protein identities appear to contribute to the mechnosensitivity of the heart mostly by indirect criteria. Sixteen are listed in a recent review [11] that includes Ca^2+^ channels, Na^+^ channels, K^+^ channels, nonspecific-cation channels, and Cl^−^ channels. Below, we will describe and discuss two types of MS channels of known identity, of clear biomechanical properties, of resolved atomic structure, and with strong connection to cardiac functions: TRPs and K_2p_’s.

## 3. Transient receptor potential channels (TRPs)

The name of this superfamily of channels was coined upon its first discovery from a mutant blind fly, the electroretinogram of which shows a receptor potential not sustained during light pulse [16]. TRPs are found in all eukaryotes including yeasts [17]. In mammals, there are over 33 genes that encode TRP subunits, which form a superfamily of channels, classified by sequence similarity into TRP-C, -V, -M, -P, -ML, -N and -A subfamilies [18]. They have wide tissue distributions and serve varied functions. As channels, they pass cations, including the functionally important Ca^2+^. As transducers, they are polymodal, each responding to multiple stimuli. Perhaps the best know is TRPV1, (TRP vanilloid type 1), the “pepper channel” that activates at temperature above 42°C [19]. As an example of polymodality, TRPV1 is also activated by low pH, endocannabinoid, polyunsaturated acid, and other proinflammatory agents. Phosphoinositides, including PIP_2_ desensitizes it. TRPV1 has been purified and reconstituted into lipid bilayer devoid of other proteins. The reconstituted channels retained the properties described above, indicating that these properties originate from this protein and the surrounding lipids alone, with no need of other co-factors, big or small [20]. TRP channel has a general structure similar to voltage-gated K^+^-, Na^+^−, or Ca^2+^-specific channels, each comprising four subunits. In Kv, each subunit includes six transmembrane α helices called S1 through S6. S1-S4 form a peripheral domain with a main function of a voltage sensor. S5 and S6 form the core domain, which houses the ion filter towards the outer side and the inner gate at the inner end, resulting from the convergence of the four S6’s. An atomic structure of TRPV1 with a 3.4 Å resolution of the transmembrane portion has recently been obtained by cryo-electron microscopy [21]. Among other differences from the canonical voltage-gated K^+^ channel are elaborations of structures around the inner gate. The long S4-S5 linker between the voltage sensor and the pore domain lies nearly flat. The pore’s inner helix, S6, is immediately followed by the amphipathic “TRP-domain” helix, which bonds to both the S4-S5 linker and the pre-S1 helix. This entire assembly is located at the level of the inner hydrophobic-polar interface of the lipid bilayer, at which the innate lateral tension is focused. (See below). The basic structures of other TRP channels are expected to be similar. For a review of the involvement (direct or indirect) of various MS TRP channels in cardiovascular physiologyand pathology see [22].

## 4. TRPV4 (TRP Vanilloid Type 1)

Over ten types of putative MS TRP channels are expressed in the heart [22]. Their effects may or may not be direct. For example, *trpV1^−/−^* knock-out mice seem to be protected from cardiac hypertrophy due to pressure overload [23]. Here, the protective effects are likely the downstream consequence of complex events with TRPV1 being one of the many participants. TRPV4, however, responds directly to physical force.

TRPV4 is broadly expressed [24] and there are human TRPV4 mutants with bone-development or other phenotypes [25]. It clearly functions in the heart. For the mechanoelectric feedback (MEF), the stretch of the heart must be detected by nerve that attaches to it. In a recent study, Shenton and Pyner (2014) [26] examined atrial endocardium using anti-synaptophysin antibody to mark the nerve endings and also immune-labeled nine different known MS-channel proteins of the ENaC/ASIC or the TRP family. They found only the immunoreactivity of TRPV4 and TRPC1 (another MS channel, see below) to precisely coincide with that of synaptophysin. This coincidence strongly suggests that TRPV4 and TRPC1, and not other putative MS channels, transmit the information of endocardial stretch to nerve endings.

Vasoconstriction is a major mechanism that controls blood pressure. The smooth muscle of the blood vessels constrict or dilate according to the forces of flowing blood detected by the endothelium. A phenomenon known as “flow-mediated dilation” [27] relates the shear stress on the endothelium to smooth-muscle dilatation through two classes of mechanisms: (1) the release of local regulators such as nitric oxide and prostacyclin to relax the smooth muscle, and (2) hyperpolarization spreading from the endothelium to muscle through gap junction [28]. This flow-mediated dilation is greatly reduced in *trpV4^−/−^* mice [29]. Current notion is that shear on the endothelial cells mechanically open channels, including TRPV4, to let in Ca^2+^, which causes the endothelium to release soluble factors to hyperpolarize the membrane of the adjacent vascular smooth muscle cells [30]. In rat carotid artery endothelial cells, a TRPV4-specific agonist, 4αPDD, causes robust endothelium-dependent vasodilatation [31]. The Ca^2+^ entered through the MS TRPV4 can activate Ca^2+^-dependent K^+^ channels (IK, SK) and cause an endothelial hyperpolarization, which can spread electrically through gap junction to the neighboring smooth-muscle cells. Sonkusare *et al.* (2012) [32] expressed the genetically encoded Ca^2+^ biosensor, GCaMP2, exclusively in vascular endothelium, and found that TRPV4-specific agonists GSK1016790A and 4αPDD to trigger fluorescent “sparklets” (elementary unitary Ca^2+^-induced fluorescent signals). Patch-clamp experiments showed that the agonist-induced currents in these endothelial cells are blocked by toxin targeting IK or SK. In short, recent findings indicate shear stress mechanically open TRPV4, through which the entered Ca^2+^ initiates outward current that spreads into and helps relax the neighboring smooth muscle.

**Fig. 1:**
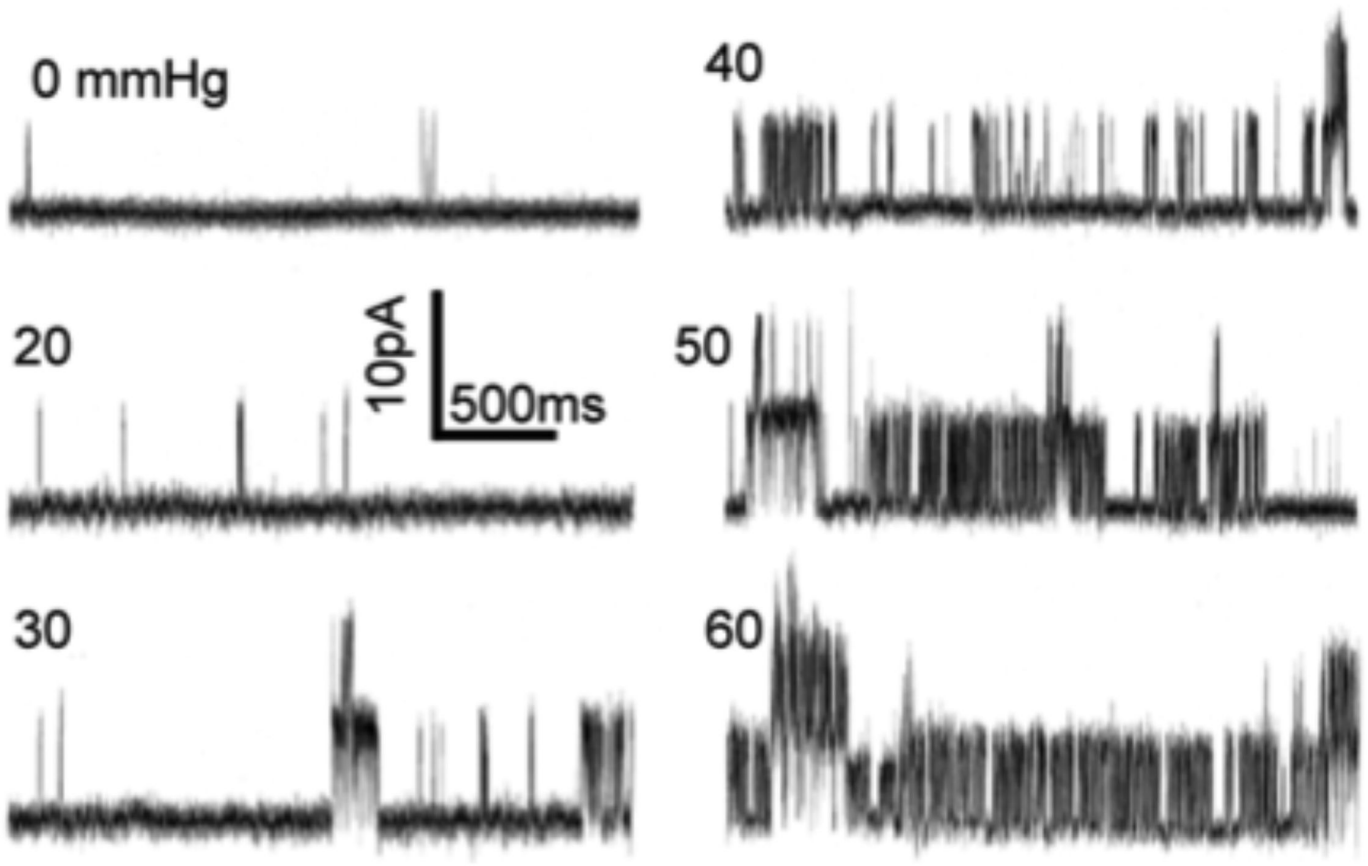
Direct mechanosensitivity of TRPV4 as shown by the activation of the 98-pS unitary conductance by membrane stretch under a patch clamp. Rat TRPV4 cRNA was expressed in *Xenopus* oocytes. An excised patch, held at +50 mV, bathed in symmetric K^+^ solution, was subjected to pipet suction, as marked, measured with a manometer. From Loukin *et al.* (2010) [33]

When expressed in *Xenopus* oocyte, bath hypo-osmolarity swells the oocyte and activates the macroscopic current of TRPV4. Inside-out patches excised from such oocytes show that the open probability of the 98-pS unitary conductance of TRPV4 increases directly with pipet suction (Fig. 1) [33]. An earlier model states that hypo-osmolarity activates enzymes (phospholipase A_2_ and P450 epoxygenase), producing a special polyunsaturated fatty acid (PUFA), called 5’6’-epoxyeicosatrienoic acid, which opens TRPV4 [34]^,^ [35]. This may be an amplification loop since Ca^2+^-entered through TRPV4 can activate phospholipase A_2_ and PUFAs alter the force distribution within the bilayer (see below). Interestingly, rat TRPV4 can be expressed in the budding yeast and still activates upon hypo-osmolarity [36]. Since attaching the rat channel to toad or yeast cytoskeleton is unlikely, the gating force for TRPV4 most likely comes from the membrane.

Of the six members of the TRPV family, TRPV4 show highest sequence homology to TRPV1, of which we now have atomic structures. This makes hopeful the sub-molecular understanding on how mechanical force operates the TRPV4 protein.

## 5. TRPC’s (“TRP Canonical”s)

Several members of the TRPC family channels, especially TRPC1, TRPC3, and TRPC6, are considered mechanosensitive and relate to cardiac function [11] [10]. Maroto *et al.* (2005) [37] detergent-solubilized frog oocyte membranes and followed MS-channel activities in fractions upon reconstitution into liposome. They identified fractions enriched with TRPC1. Further, human TRPC1 expressed in *Xenopus* oocytes markedly increases stretch-induced current under patch clamp. Although not without controversy [38] [39], TRPC1 remains an important target in the study of cardiac physiology and pathology. In an induced rat-heart hypertrophy model, the expression of TRPC1 rises in the hypertrophic myocardium. The up-regulation of TRPC1 appears to contribute through its mechanosensitivity [40]. Furthermore, deletion of the *trpc1* gene in mice led to protection from cardiac hypertrophy, which is surprising given that the mouse heart expresses at least four other TRPC channels. However, because of its feature of stretch activation it is likely the TRPC heterotetrameric complexes present in the mouse heart require at least a single TRPC1 subunit [5]. As described above, a most recent report shows that TRPC1 and TRPV4, but not other putative MS channels reside at the endings of the nerves that innervate the atrial endocardium [26].

TRPC6 has apparently a role in sensing intravascular pressure. Antisense oligonucleotides to TRPC6 attenuate arterial smooth muscle depolarization and constriction caused by elevated arterial pressure [41]. TRPC6 channels expressed in HEK cells are activated by hypo-osmolarity. Those in patches excised from expressing cells are activated directly by pipet suctions, which can be blocked by the spider toxin GsMTx-4, known to act on the lipid-channel interface [42]. That the expressed TRPC6 can be activated by induced phospholipase C (PLC) activity or by addition of the membrane-permeable diacylglycerol (DAG) analog OAG further support the notion that TRPC6 receives mechanical force from the lipid bilayer [43].

The “C” in “TRPC” stands for “canonical”, meaning that it represents the founding member of the entire TRP superfamily. As stated above, the founding member was discovered by tracing the molecular defect of a blind fly [16]. Mutations of other blind flies revealed the key role of lipid metabolism in fly phototransduction. One key gene encodes phospholipase C (PLC), which hydrolyses PIP_2_ into diacylglycerol (DAG) and IP_3_. Neither IP_3_ nor DAG, however, can be shown to activate the TRP channels as ligands, however. Instead, Hardie and Franze (2012) [44] showed that light induces a micrometer shrinkage of each unit of the compound eye, which contains thousands of microvilli housing the phototransduction complexes. Apparently, these canonical TRP’s are MS channels, responding to the sum of the mechanical changes upon the beheading of PIP_2_ into DAG in the inner leaflet of the bilayer. These authors coined the term “photomechanical responses”. Such responses have their counterparts in mammalian photosensitive ganglion cells, and possibly even in melanocyte or keratinocyte (See [45] [46]for reviews). Suffice it to say here that bilayer-based force transmission to the heart-relevant TRPC1, TRPC6, and TRPV4 has deep evolutionary roots.

## 6. Two-pore-domain K^+^ channels (K_2p_’s)

Over ten types of K^+^ channels are expressed in different parts of the heart. Since K^+^ efflux accounting for the down stroke of action potential is well known, voltage-gated K^+^ channels have been the center of attention in cardiology. More recently, two other types of K^+^ channels have entered the picture: Ca^2+^-activated K^+^ channels (K_ca_’s) and the two-pore domain K^+^ channels (K_2p_’s ) [47].

In general, K_2p_’s are considered the background leak K^+^ channels that determine cell’s resting potentials. mRNAs of six subtypes of K_2p_’s are found in the heart. K_2p_3.1 (TASK-1/*KCNK3*) is a current research focus because *K_2p_3.1^−/−^* knockout mice or knockdown zebrafish produce ventricular or atrial phenotypes [48]. Further, several K_2p_3.1-specific inhibitors are available to be developed into antiarrhythmic drugs [47]. Interestingly the *K_2p_3.1^−/−^* mice, besides having a prolonged QT interval, also have a diminished baroreflex [49]. Baroreflex is a negative feedback that makes short-term adjustment of blood pressure, which is monitored by stretching the mechanosensitive nerve endings (baroreceptors) in major arteries.

K_2p_2.1 (TREK-1) is expressed in both the atria [50] and ventricles [51]. Extensive research has shown that TREK-1 and threlated TRAAK are mechanosensitive [52] [53]. In excised patches, TREK-1 is opened by either pipet suction or pressure, indicating that it is the lateral stretch of the patch that activates. It is also activated by lysophospholipids, PUFAs, PIP_2_, and various lipid-soluble anesthetics [52] [53]. Immunohistochemistry and patch-clamp experiments showed that TREK-1 is expressed in cardiomyocyte membrane and may contribute to mechanoelectric feedback and arrythomogenesis [54]. Though the matters seem complex, down regulating K_2p_2.1 in right atrial tissue in a porcine model correlates with atrial fibrillation and heart failure [47].

**Fig 2:**
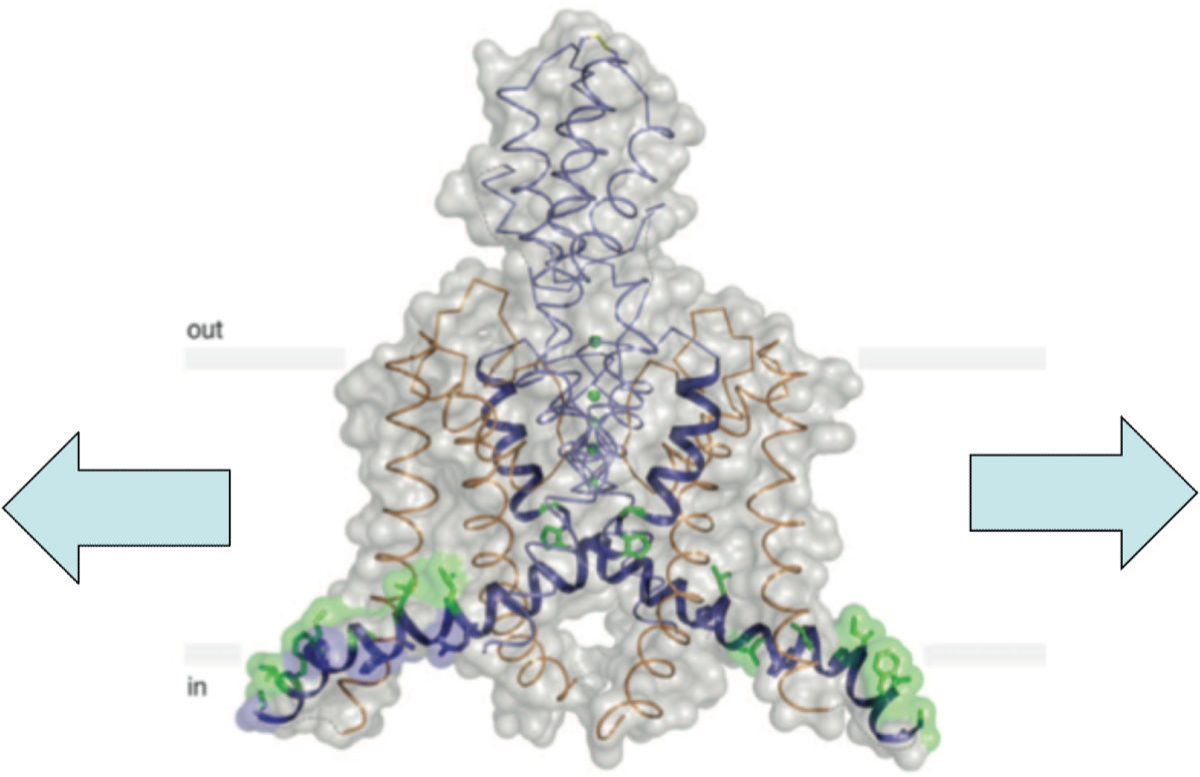
Crystal structure of TRAAK, a mechanosensitive K_2p_ channel. For a description see text and Brohawn *et al.* (2012) [55]. Purified TRAAK, reconstituted into lipid bilayer, retains its response to added bilayer stretch (arrows) (Brohawn *et al*., 2014) [56]. Modified from Brohawn *et al*. (2012) [55].

The simplest K^+^ channels are tetramers of subunits with a S_1_-P-S_2_ arrangement. Others, however, are tetramers of S_1_-S_2_-S_3_-S_4_-S_5_-P-S_6_ subunits, where the S’s are transmembrane helices and P the filter structure. K_2p_’s, however, comprises two S_1_-P_1_ -S_2_-S_3_-P_2_-S_4_ subunits and therefore has a 2-fold instead of 4-fold symmetry. The two S_2_’s and the two S4’s encase the filter and line the ion pathway. The crystal structures of two K_2p_ channels have recently been solved [55,57]. The one of TRAAK, resolved at 2.75 Å, is of particular interest here (Fig. 2) [55], [58]. TRAAK is similar to TREK-1 in sequence and is known to be mechanosensitive [59]. Among the unique features of TRAAK are the two S2 helices, which are long and each has a kink that makes the lower portion lying almost flat. This portion is clearly amphipathic with hydrophobic residues facing the membrane interior and hydrophilic basic amino acids facing the membrane-cytoplasm interface. This is the level at which the bilayer’s innate lateral tension is focused (see below). The inner half of TRAAK, unlike most other K^+^ channels, is fenestrated, meaning that the ion pathway is not completely enclosed with peptides, allowing bilayer lipids direct access to that pathway.

## 7. The Force-From-Lipid (FFL) paradigm (Fig. 3)

For some 25 years, there have been two models on how physical force opens MS channels: First, based entirely on biophysical properties and not on biochemical material, the vertebrate hair-cell transduction channel has early on been modeled to be like a trapdoor. To explain compliance, the trapdoor is modeled to have a mechanical gating spring, which maps to the anatomically visible tip link [60]. More recently, the tip-link proteins (cadherin-23 and protocadhedrin-15) and other accessories have been substantiated, but the identity of MS transduction channel itself remains elusive, despite several false starts [61]^,^ [62]. Some other eukaryotic models may also employ external and/or internal tether to transmit force. The touch receptor channel of the worm *C. elegans*, for example, has been modeled to use cytoskeletal microtubules to transmit force. Yet genetically removing the tip link or the microtubules greatly reduces but does not remove the MS channel’s response to force [63,64]. External or internal tethers may be amplification devices. They may tense up the membrane around the MS channel by pulling on the channel or the membrane. See [46] for a review.

The second model began with the discovery of MS channels of *E. coli* [65]. The pentameric membrane protein MscL, made up of five 136-amino-acid subunits, has repeatedly been purified to homogeneity and reconstituted into bilayers of known lipids and found to retain its mechanosensitivity [66,67]. The reductive nature of such experiments leaves the lipid bilayer as the only possible source of mechanical force. Further, amphipaths that preferentially intercalate into one of the leaflet can activate MscL and its functional analog MscS [68] [69] [70]. Crystal structures, genetic dissections, spectroscopy and other physical analyses made MscL and MscS the key models for detailed mechanistic understanding of molecular mechanosensitivity [69,71]. However, MscL and MscS are often viewed as bacterial specialization. This view is now challenged by the recapitulation of the same reductive experiment with two vertebrate MS channels. Berrier *et al*. (2013) [72] have reconstituted an enriched fraction of the mouse TREK-1 into liposome and showed the K^+^ unitary conductances to respond to force under patch clamp. In a set of more rigorous experiments, Brohawn *et al.* (2014) [56] purified zebrafish TREK-1 and human TRAAK to homogeneity and reconstituted them to azolectin bilayers. So treated, these channels in excised patches continue to respond to bilayer tension. Note that TRAAK is now of known molecular structure (see above and Fig. 2). It is therefore at an experimentally advantageous position that parallels those of MscL and MscS.

**Fig 3:**
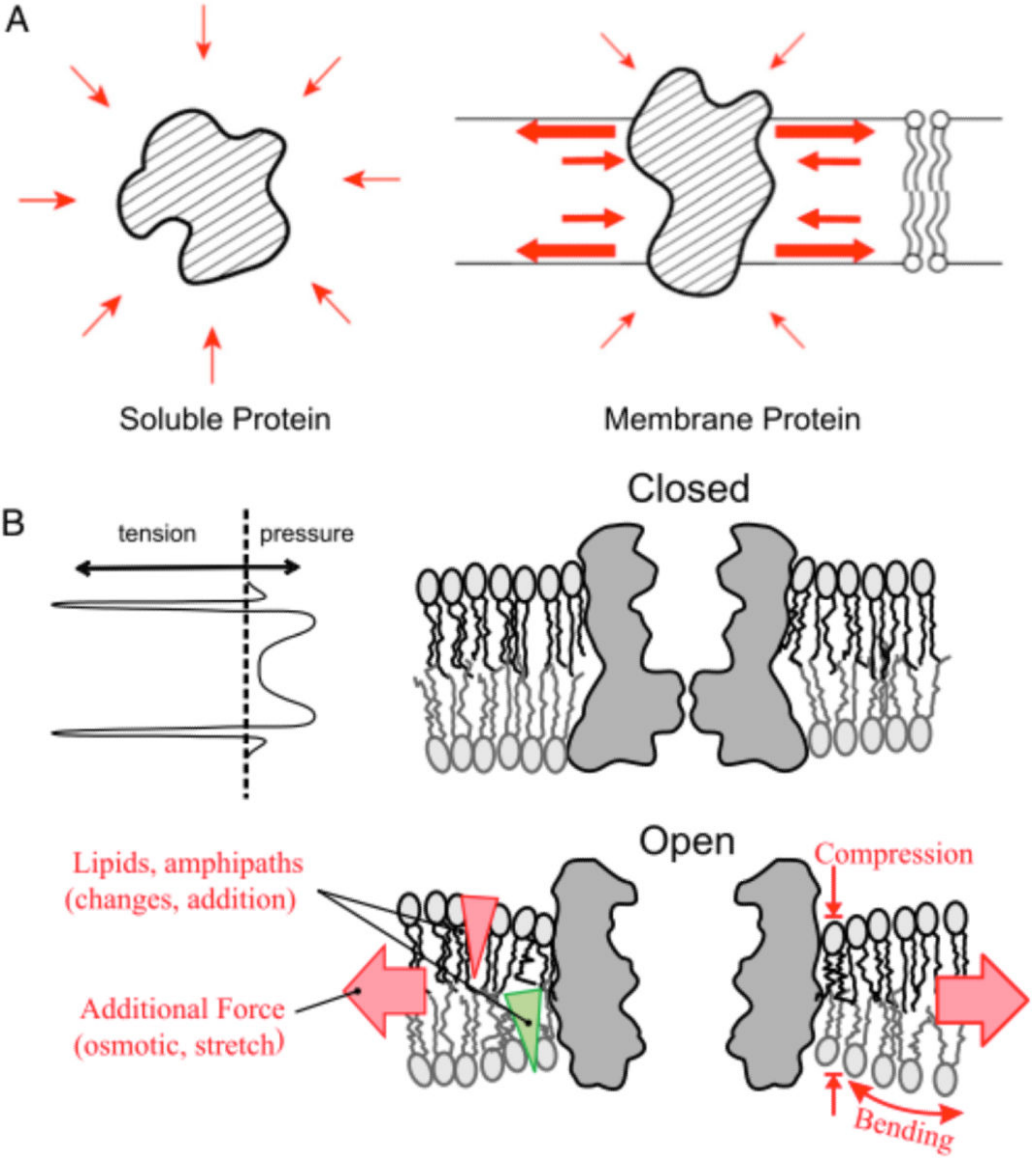
The force-from-lipid (FFL) principle. (A) Unlike a soluble protein, bombarded by neighbors(left), a membrane protein (right) is embedded in the lipid bilayer, which has standing internal forces (red arrows). (B) (upper left) The profile of these forces shows peaks of lateral tension at the two polar/nonpolar junction, at the level of the lipid neck. At rest, a MS channel (upper right) can be in a closed state with its surface residues matching this profile. When the bilayer is stretched (broad red arrows, in lower figure) or when amphipaths (triangles) are added, the bilayer is deformed (compressed and/or bent), and the channel can be energetically driven into the open conformation to better match the lipids. From Anishkin *et al.* (2014) [45].

To see how lipids gate MS channels, one begins with the structure of the bilayer, which is not derived from genetic information, but by thermodynamics. To avoid the energy cost of entering the non-polar region, the polar moieties (water, ions, *etc*.) congregate at any water-lipid interface and try to minimize its area, thus resulting in a surface tension. A similar lateral tension develops in the bilayer at the level of the lipid neck between the polar heads and the non-polar tails. Bilayer is a self-assembled stable structure, and the area compaction is stopped when this lateral tension is balanced by repulsions elsewhere at the head and the tail regions (Fig. 3). Any material, such as membrane proteins, embedded in the bilayer, is subjected to these forces, the sharp lateral tensions in particular. At equilibrium, an embedded protein will be at a conformation, say, the channel closed state, presenting a shape and a surface polarity that optimally matches this profile. Added external forces can stretch and thin and/or bend the bilayer, changing the force profile. Adding lipids or amphipaths, especially asymmetrically into one leaflet, will also change this profile [70]. The protein may no longer match the new profile and is therefore energetically driven to a better-matched conformation, say, the open state (Fig. 3B), typically complying in the direction of the net force. MscL opens by outward movement and the leaning of its helices, increasing its lateral area about twice, and resulting in a much thinner ring, as if to meet a thinned bilayer [70]. In detail, residues or local domains differ in their affinity to different lipids at the interface [73], and the binding of lipids will likely distort the bilayer arrangement [74]. Regardless of details, added force, such as a stretch, will change the magnitude and direction of the force vectors acting on the protein through the bonds at the interface. For more detailed explanation of the force-from-lipid (FFL) principle, see [75] [76] [46]. All cells are wrapped in membranes comprising lipids, the assembly of which is driven by thermodynamics. One would therefore expect FFL-based mechanosensitivity to have arisen near the beginning of life and therefore likely to underlies all forms of force sensitivities and sensations evolved later [76].

## 8. Extension and conclusion

The increasing number of crystal or cryo-electron microscopic structures of membrane proteins has greatly increased our knowledge. Because of the dynamic liquid nature of biological membranes and because detergents instead of lipids are used to purify these proteins, these structures usually only show the amino acids. This can be misleading. When lipids are included in the crystal, as in the case of the voltage-gated K^+^ channel [74], some are clearly bound to the amino acids, held tight enough to be seen at atomic resolution. When a channel, such as Kv, opens, the free energy change in rearranging the lipids in the bilayer is estimated to be comparable to that of the voltage-dependent part of the total gating energy [77]. *In situ*, each membrane protein has strongly bonded annulus lipids and the entire ensemble is re-configured during a conformational change.

We have reviewed the bilayer, its standing force profile, and the FFL principle of MS-channel gating above. Given the view that a channel, or any membrane protein, is in fact a protein-lipid ensemble, does that mean they all feel the bilayer force? The answer, which may surprise some, is yes. Yet, to state that all membrane proteins are mechanosensitive should not be more surprising than to say all proteins, indeed, all material, are heat-sensitive. While such universal mechano- or heat-sensitivity is a physical truism, whether they are used biologically is a different matter.

By various criteria, many channels, including K^+^-, Na^+^-, Cl^−^-,or ion-nonspecific channels, known to be gated by voltage, by Ca^2+^, or by other means, have been reported to also be sensitive to mechanical or osmotic forces. Some of them clearly function in the heart. See [75] for a list. In a set of careful experiments and analyses, the canonical voltage-gated K^+^ channel is found to behave drastically differently in on-cell patches, where the bilayer is restrained by cortical cytoskeleton, in excised patches, where the cytoskeleton is detached, and after reconstitution into bilayer, which is pre-stretched by the lipid-glass adhesion [78]^,^ [79]. The different behavior is clearly correlated to membrane stretch force. Kinetic analyses showed that the tension-sensitive step is at the point of channel expansion, after all four voltage sensors have moved. Quantitative comparison shows that Kv is as much a mechano-sensitive channel as a voltage-sensitive channel [79]. Our preconceived notion may mislead us to regard such channels as primarily voltage-gated channels, but only secondarily “modulated” by mechanical force. A recent report shows that prodding certain dorsal-root-ganglion neurons activates a K^+^ current through channels containing Kv1.1 subunits. Such an outward current seems to act as a brake against the touch-induced inward currents through other MS channels. Interestingly, mice expressing a dominant negative Kv1.1 subunit are pathologically over-sensitive to touch [80]. While this one startling case seems clear, it remains to be seen whether the mechanosensitivity of other K^+^-, Na^+^-, Cl^−^-,or ion-nonspecific channels are physiologically meaningful. Nevertheless, one should not only evaluate the mechanical activation of channels in mechano-electric feedback reviewed above, but also be prepared to re-evaluate the mechanical activation of those channels responsible for pace making and in EC coupling in the constantly beating heart.
